# Comparing conventional versus 3D printed simulators for simulation training of emergency percutaneous cricothyrotomy with two different kits: a randomized controlled trial

**DOI:** 10.1186/s41205-026-00315-z

**Published:** 2026-02-02

**Authors:** Moritz Wegner, Fabian Dusse, Finnard Beeser, Nicolas Leister, Marian Lefarth, Simon-Richard Finke, Bernd W. Böttiger, Andrea U. Steinbicker, Bernhard Dorweiler, Sandra Emily Stoll

**Affiliations:** 1 Department of Vascular and Endovascular Surgery, Faculty of Medicine and University Hospital Cologne, University of Cologne, Cologne, Germany; 2 Department of Anesthesiology and Intensive Care Medicine, Faculty of Medicine and University Hospital Cologne, University of Cologne, Cologne, Germany; 3 Department of Anesthesiology, Montefiore Medical Center, Albert Einstein College of Medicine, Bronx, NY USA

**Keywords:** Simulation, Simulation training, 3D printing, Airway management, Emergency medicine, Percutaneous cricothyrotomy

## Abstract

**Introduction:**

Effective procedural training is crucial for emergency interventions such as percutaneous cricothyrotomy (PC). This study evaluated simulation-based training of PC by comparing two simulators, a commercially available conventional simulator (CSIM) and an innovative 3D-printed simulator (3DSIM), and assessed their impact on procedural performance and subjective safety perceptions using two different PC kits: Quicktrach II (direct puncture method) and Surgicric III (Seldinger technique).

**Methods:**

Forty-four participants underwent standardized theoretical training and were randomized into two groups: Group A initially trained with CSIM and Group B with 3DSIM. In both groups, procedural performance was evaluated immediately after each simulation session on porcine trachea models by two blinded assessors. Outcomes included procedural time, standardized performance scores, and subjective safety ratings. Participant evaluations of educational benefit and simulator realism were also recorded. Training effectiveness was reassessed in a second session using a crossover design, allowing direct comparison of the two simulators and kits.

**Results:**

Procedural performance improved significantly after repeated training, with no significant differences between CSIM and 3DSIM regarding procedural times (*p* = 0.98) or accuracy scores (*p* = 0.99). Both PC kits showed significantly reduced procedural times (Quicktrach II: 42 ± 46 to 19 ± 7 s, *p* < 0.01; Surgicric III: 119 ± 73 to 91 ± 51 s, *p* = 0.03). Accuracy improved significantly only for Surgicric III (95 ± 10% to 98 ± 4%, *p* = 0.04). Participants’ perceived safety improved similarly for both simulators (44 ± 20% to 94 ± 9%), without differences in educational benefit or realism ratings.

**Conclusion:**

Conventional and 3D-printed simulators were equally effective in enhancing procedural performance and subjective safety perceptions in PC training. Procedural time improvements differed by kit complexity, suggesting Quicktrach II offers quicker initial mastery, whereas Surgicric III may require additional practice due to its greater complexity. These results support flexible simulator choice based on local factors like cost and availability, underscoring the potential of 3D-printed simulators for procedural training programs.

**Clinical trial number:**

Not applicable.

**Supplementary Information:**

The online version contains supplementary material available at 10.1186/s41205-026-00315-z.

## Introduction

Difficult airway scenarios requiring an emergency surgical airway access are rare but potentially life- threatening events that demand rapid and competent intervention. In ‘cannot intubate, cannot ventilate’ situations, percutaneous cricothyrotomy (PC) is recommended as a last- resort rescue technique, as outlined in the guidelines by the American Society of Anesthesiologists [1]. Despite its critical importance, PC is infrequently performed in routine clinical practice, leading to limited procedural experience among healthcare professionals. This lack of exposure is reflected in findings from the Fourth National Audit Project (NAP4), where 60% of emergency surgical airways using PC failed, indicating both technical challenges and potentially inadequate training in real-life scenarios [2]. These data underscore the need for targeted training approaches to improve preparedness and procedural proficiency.

Simulation-based medical education (SBME) has emerged as a valuable tool to enhance confidence and competence in rare but high-stakes interventions by offering structured, risk-free environments for hands-on learning [3]. While especially pertinent for high-acuity fields such as anesthesiology and emergency medicine, the benefits of SBME extend across medical disciplines, particularly in training “high-acuity, low-occurrence” (HALO) procedures like PC.

Various training models for PC have been described, including embalmed cadavers, porcine tracheas, commercial simulators, and more recently, high-fidelity 3D-printed models. However, few studies have directly compared different simulator types using standardized performance metrics. [4–7] Likewise, limited data exist comparing different commercially available PC kits with distinct techniques, such as the Quicktrach II (puncture-based, VBM Medizintechnik GmbH, Sulz, Germany) and the Surgicric III (Seldinger technique, VBM Medizintechnik GmbH, Sulz, Germany) under controlled simulation conditions.

This study aims to address these gaps by comparing the impact of two simulator types, a commercially available conventional simulator (CSIM) and a high-fidelity 3D-printed simulator (3DSIM), on PC training using both kits. Our primary objective was to evaluate procedural performance, measured by time and accuracy. Secondary objectives included subjective safety perception and user evaluation of the educational value and realism of each simulator.

## Materials and methods

### Study design

We performed a prospective, randomized, blinded, single-center crossover study comparing the efficacy of simulation training of PC with two simulator types: a commercially available conventional simulator (CSIM) and a high-fidelity 3D-printed simulator (3DSIM). Participants used two different PC kits (Quicktrach II and Surgicric III). The choice of kits was based on local availability, as these two kits are routinely used at our institution and represent two distinct procedural techniques (Quicktrach II employs a direct puncture method, while Surgicric III uses the more complex Seldinger technique). The simulator types were selected to evaluate the potential advantages of innovative 3D-printing technology in procedural training compared to an established, commercially available model. Each participant underwent standardized self-paced online theoretical training lasting 30 min and completed a questionnaire covering demographic details such as age, sex, the professional experience, and the number of prior PC procedures. Participants also rated their theoretical knowledge, number of previously performed PC and their perceived grades of safety of performance of a PC on a 5- point Likert scale.

After theoretical training, 50 participants were planned based on a sample size calculation using Student’s t-test (two-tailed), a statistical power of 80%, alpha error of 0.05, and an anticipated effect size of < 0.4. Randomized allocation was performed by a prepared envelope system created by a person not involved in the study setting. Participants were randomly assigned to an initial standardized simulation training session lasting 20 min using either (A) commercially available simulators (CSIM) or (B) 3D-printed simulators (3DSIM), during which both groups trained with two different PC kits (Quicktrach II and Surgicric III). Following initial simulation training, participants’ PC performance was immediately evaluated by two independent assessors who were blinded to simulator type, PC kit type, participant training group assignment, and assessment round. Assessments were performed directly on porcine trachea models without video recording. Procedural timing was strictly defined, beginning when participants first touched the PC kit and ending when airway access was confirmed (air aspiration via syringe for Quicktrach II or insertion of the Seldinger wire for Surgicric III). The accuracy of PC was assessed using a predefined scoring system (Quicktrach II, maximum 11 points; Surgicric III, maximum 19 points; see SDC Tables 1 and 2). Subsequently, participants completed a post-training questionnaire, assessing subjective safety regarding future PC performance, subjective educational benefit, and perceived optical and haptic realism of the simulator using a 5-point Likert scale.

Afterwards, a second PC simulation training session was conducted under identical conditions in a crossover design, with group (A) initially training with CSIM now training on 3DSIM, and group (B) initially training on 3DSIM now training on CSIM. A second blinded assessment of PC performance on the porcine larynx followed immediately, using the same criteria.

The training protocol and assessments for each group were explicitly as follows:

Group A.

1. First Training Session: Participants trained on the CSIM simulator. During this session, they first trained using the Quicktrach II kit (10 min), followed by the Surgicric III kit (10 min).
2. First Assessment: Participants were evaluated on a porcine trachea by 2 blinded examiners. Assessment 1 consisted of two evaluations:

- Performance using the Quicktrach II kit.
- Performance using the Surgicric III kit.
  3. Second Training Session: Participants trained on the 3DSIM simulator. The sequence of training mirrored the first session: Quicktrach II kit training (10 min) followed by Surgicric III kit training (10 min).

4. Second Assessment: Participants were again evaluated on a porcine trachea, with the same sequence:

- Performance using the Quicktrach II kit.
- Performance using the Surgicric III kit.

Group B.

The protocol was identical to Group A, except for the order of simulators:

1. First Training Session: Participants trained on the 3DSIM simulator, using the Quicktrach II kit first, followed by the Surgicric III kit.
2. Second Training Session: Participants trained on the CSIM simulator, following the same sequence as the first session.
3. Assessments 1 and 2 followed the same structure as Group A, with evaluations on the porcine trachea using both kits.

**Fig. 1:**
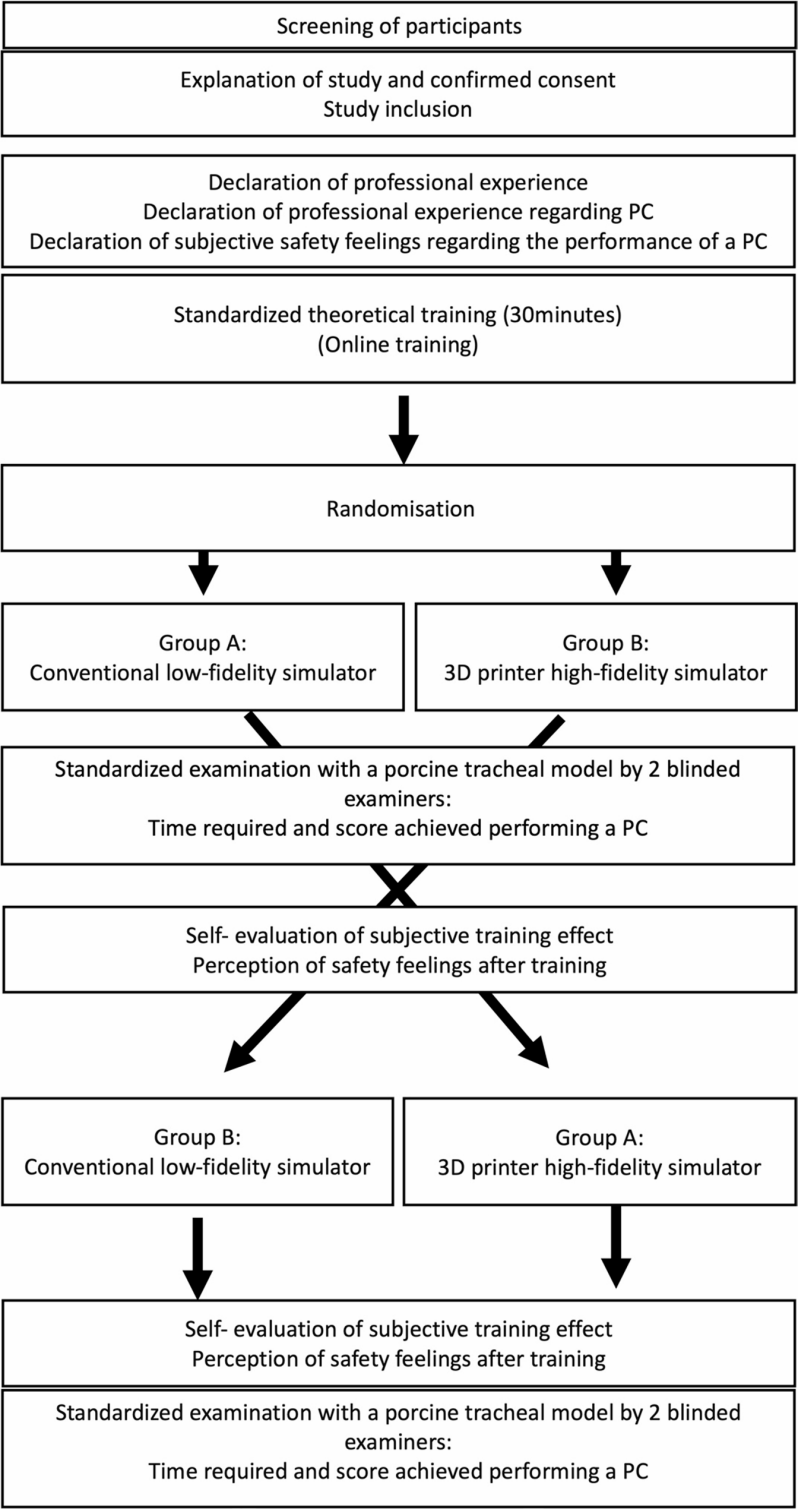
Flowchart of the study protocol

Upon completion, participants provided final feedback via questionnaires, rating their perceived safety and their educational benefit. Figure 1 depicts a flow-chart of the study protocol.

### Percutaneous cricothyrotomy systems

The study compared two distinct percutaneous cricothyrotomy (PC) kits: Surgicric (III) and Quicktrach (II) (both manufactured by VBM Medizintechnik GmbH, Sulz, Germany). Surgicric III utilizes Seldinger’s technique, while Quicktrach II employs a 4 mm diameter puncture cannula for airway access.

### Commercially available simulators (CSIM)

**Fig. 2:**
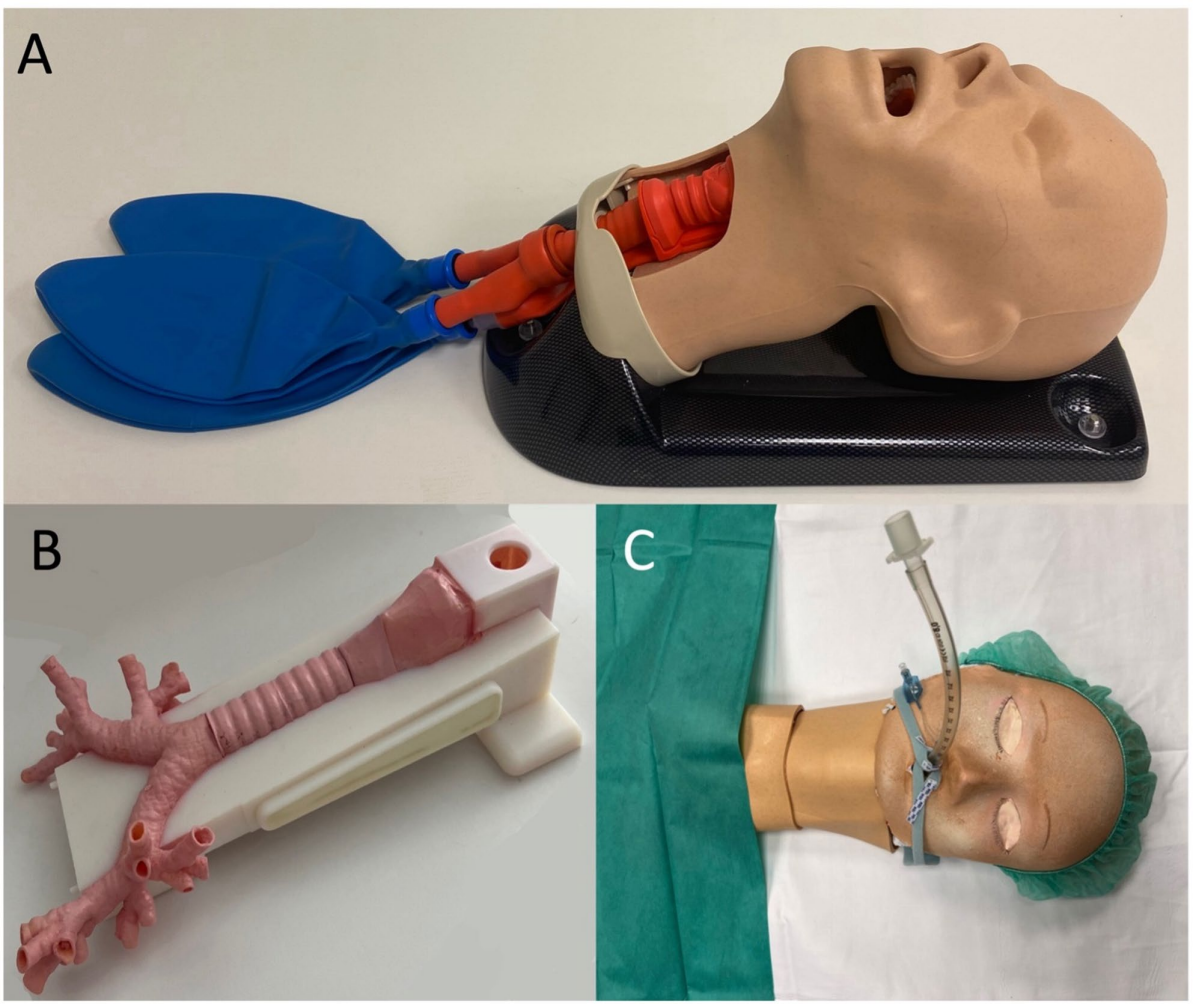
Different study simulators (**A**: conventional simulator, **B** and **C** 3D printer generated simulator)

To conduct simulation training with CSIM, we utilized the AirSim Combo X^®^ (TruCorp Ltd., Lurgan, United Kingdom), a simulator featuring a realistic 1:1 scale representation of an adult head including the airway (larynx, trachea, bronchi) with palpable cricoid landmarks, laryngeal cartilages and tracheal rings. For a more realistic setting the larynx and the trachea were covered with a silicone skin flap imitating natural skin (see Fig. 2A).

### 3D printed simulator (3DSIM)

As previously described, we produced an accurate, life- sized replica of the human laryngo- tracheo- bronchial system using 3D engineering software and printing technology [8]. Utilizing specialized software (Mimics Innovation Suite, Materialise, Belgium), we extracted this system from anonymized CT scan data of the neck and the chest acquired at our institution. Initially, anatomical structures like the larynx, including the epiglottis, the vocal cords, the thyroid cartilage, and the cricoid cartilage, were semi-automatically segmented on the CT images using the “region growth” tool. Subsequently, the tracheobronchial air volume was segmented, and a tracheobronchial wall with a 2 mm thickness was added to the intratracheal air volume using the “hollow” tool of the design software (3- Matic, Materialise, Belgium). Tracheobronchial ostia were then trimmed to the subsegmental level, and the trachea was designed as a separate removable part, facilitating the replacement after potential damage during simulated procedures. Additionally, the same software was employed to create a socket for securing all anatomical components and providing a stable base for the model. Following these steps, a 3D file (.stl) was generated and printed using a high- resolution PolyJet- technology 3D printer (Objet 350 Connex3, Stratasys, Rechovot, Israel). The model included both, flexible parts (larynx, trachea, and bronchial system) and the socket, utilizing Agilus^®^30 and VeroWhite^®^ materials. Printing resolution was maximized with a layer resolution of 16 μm. After printing, thorough cleaning and removal of support materials were performed. Previous research has demonstrated that this process yields 3D- printed models with high anatomical fidelity and minimal spatial deviation (± 120 μm [9]. For a more realistic experience, the 3D- printed simulator (Fig. 2B) was then covered with a cardboard face mask (representing the head) and a silicone skin flap for imitation of natural skin (Fig. 2C).

### Evaluation of the PC performance on a Porcine larynx

The effectiveness of each simulation training was assessed on a porcine model by two blinded examiners checking for (1) time required for performing the PC and (2) the accuracy of PC evaluated by a predefined scoring sheet (see SDC Tables 1 and 2 in the supplement).

Two blinded examiners assessed the performance of the PC with the Surgicric III kit on a porcine trachea and two other blinded examiners assessed the performance of the PC with the Quicktrach II kit on a porcine trachea.

### Pre- and post- simulation training self- evaluation questionnaire

Each participant completed a self- assessment questionnaire, both before simulation training and after the first and second simulation training sessions. Thereby, the participants theoretical understanding and their previous experience with PC before simulation training were evaluated. The participants also evaluated the efficacy of the different simulators regarding their perceived feeling of safety before and after simulation training, the perceived learning benefit and the optic and haptic authenticity of the simulator using a 5- point Likert scale. All questionnaires and scales haven been previously used und published by our study team for evaluating simulation training [8]. 

### Statistical analysis

All statistical analyses were performed using SPSS Version 29.0 (IBM Corp., Armonk, NY, USA) and R Version 4.4.2. Data were assessed for normality using the Shapiro-Wilk test. Continuous variables were presented as means ± standard deviations or medians with interquartile ranges, depending on the data distribution. Categorical variables were presented as frequencies and percentages. Baseline characteristics were compared between Group A and Group B using independent samples t-tests for continuous variables and chi-squared tests for categorical variables. A p-value of < 0.05 was considered statistically significant. These analyses were performed without correction for multiple testing, as the focus was on ensuring comparability between groups rather than hypothesis testing.

Procedural times and standardized scores were analyzed using separate repeated- measures analysis of variance (ANOVA) models. The factors included kit type (Quicktrach II vs. Surgicric III) and assessment (first vs. second) as within- subject factors and group assignment (Group A vs. Group B) as a between- subject factor. Interaction effects between these factors were also evaluated.

Post- hoc comparisons were performed using estimated marginal means (EMMs) with Tukey’s HSD adjustment to account for multiple comparisons.

For the analysis of personal grades of safety across the three time points (before the first simulation training, after the first training, and after the second training), a repeated-measures ANOVA was conducted with time as the within-subject factor and group (A vs. B) as the between-subject factor. Post- hoc analyses were performed using pairwise comparisons with Tukey adjustments. Sphericity violations were addressed using the Greenhouse- Geisser correction. All visualizations were created using the ggplot2 package in R.

### Ethical approval

The study received approval from the Ethical Committee of the University of Cologne (IRB 22-1141_1). All participants provided written informed consent before the study inclusion, adhering to the principles outlined in the Declaration of Helsinki.

## Results

In this study, 44 of 50 participants were included (comprising 40 physicians and 4 medical students). Six participants gave their informed consent and received theoretical training but withdrew from simulation training in the day of the training due to personal reasons. These participants were randomized to one of the groups before their withdrawal. Of the 44 participants, 24 initially trained with CSIM (group A), while 20 initially trained with 3DSIM (group B). In group A 22 physicians and 2 medical students participated in simulation training versus 20 physicians and 2 medical students in group B. Analyses were performed according to the initially assigned group.

### Demographic data

The baseline characteristics were comparable between the groups and no significant differences were observed. For comparisons of baseline characteristics, no corrections for multiple testing were applied, as these analyses were exploratory in nature and intended to describe the study cohort rather than to test specific hypotheses. A detailed summary is provided in Table 1.

**Table 1:** Baseline characteristics of *n*= 44 participants prior to the simulation training of percutaneous cricothyroidotomy (PC)

	Group A 1) CSIM 2) 3DSIM (*n*=24)	Group B 1) 3DSIM 2) CSIM (*n*=20)	Total (*n*=44)	*p*-value
Sex (male)	9 (37.5%)	10 (50.0%)	19 (43.2%)	*0.54*
Age (*years*)				0.87
*18-30*	8 (33.3%)	5 (25.0%)	13 (29.5%)	
*31-45*	15 (62.5%)	14 (70.0%)	29 (65.9%)	
*46-60*	1 (4.2%)	1 (5.0%)	2 (4.6%)	
Professional Experience (*years*)				0.84
* <5*	14 (58.3%)	11 (55.0%)	25 (56.8%)	
*5-10*	7 (29.2%)	5 (25.0%)	12 (27.3%)	
*10-20*	3 (12.5%)	4 (20.0%)	7 (15.9%)	
Profession				1.0
*Physician*	22 (91.7%)	18 (90.0%)	40 (90.9%)	
*Medical student*	2 (8.3%)	2 (10.0%)	4 (9.1%)	
Number of previously performed PC	0	0	0	-
Self-assessment of theoretical knowledge about PC				0.76
*None*	0	0	0	
*Low*	1 (4.2%)	3 (15%)	4 (9.1%)	
*Medium*	9 (37.5%)	7 (35%)	15 (36.4%)	
*Good*	12 (50%)	8 (40%)	20 (45.5%)	
*Very Good*	2 (8.3%)	2 (10%)	44 (9.1%)	

Data are presented as *n* (%)

CSIM= Conventional simulator, 3DSIM= 3D printed simulator, PC= percutaneous cricothyrotomy

Correction for multiple testing was not applied to the comparison of baseline characteristics, as these analyses were descriptive and intended to assess group comparability rather than to test specific hypotheses

### Procedural times and scores – first assessment

Table 2 summarizes the procedural times and standardized scores across kits (Quicktrach II vs. Surgicric III) and simulators (CSIM vs. 3DSIM) during the first assessment. The first assessment was used to primarily compare the two different simulators (CSIM and 3DSIM), as this was only possible before both groups trained with both simulators. A two-way ANOVA revealed a significant main effect of kit type on procedural time (F (1,87) = 33.24, *p* < 0.001, η²= 0.28), indicating shorter times for Quicktrach II compared to Surgicric III. However, neither the main effect of simulator type (F (1,87) = 0.00, *p* = 0.98, η²= 0.00) nor the interaction between kit and simulator (F (1,87) = 0.20, *p* = 0.65, η²= 0.002) was significant.

**Table 2:** Comparison of simulators (CSIM vs. 3DSIM) and kits (Quicktrach II vs. Surgicric III) on a Porcine laryngeal model after assessment 1 after simulation training 1

Kit	Simulator	Time (seconds)	Score (% of maximum achievable)
Quicktrach II	CSIM (*n*=24)	39 ± 39	96 ± 8
	3DSIM (*n*=20)	45 ± 55	92 ± 10
Surgicric III	CSIM (*n*=24)	122 ± 69	93 ± 13
	3DSIM (*n*=20)	115 ± 78	96 ± 5

Data are presented as mean ± standard deviation

CSIM= Conventional simulator, 3DSIM= 3D printed simulator

Regarding standardized scores, neither the main effects of Kit, F(1, 87) = 1.19, *p* = 0.19, η²= 0.01, and Simulator, F(1, 87) = 0.00, *p* = 0.99, η² = 0.00, nor the interaction between Kit and Simulator, F(1, 87) = 2.29, *p* = 0.13, η² = 0.03, were significant, indicating that procedural scores were comparable across kits and simulators. Baseline characteristics, including age, sex, professional background, years of professional experience and self- assessment of theoretical knowledge did not significantly affect procedural time or scores during the first and second assessment. Therefore, these analyses are not presented in detail.

### Improvement of procedural times and scores after second assessment

Table 3 summarizes the procedural times and standardized scores across kits and groups (A vs. B) during both assessments. This analysis was used to investigate the effect of repeated simulation training.

**Table 3:** Time and score changes for the Quicktrach II and Surgicric III kits on a Porcine laryngeal model between assessments 1 and 2 for groups A and B

Kit	Group	Time assessment 1 (seconds)	Time assessment 2 (seconds)	Score assessment 1 (% of maximum achievable)	Score assessment 2 (% of maximum achievable)
Quicktrach II	Group A (*n*=24)	39 ± 39	20 ± 7	96 ± 8	97 ± 7
	Group B (*n*=20)	45 ± 55	19 ± 8	92 ± 10	98 ± 6
	Both Groups (*n*=44)	42 ± 46	19 ± 7	94 ± 9	97 ± 7
Surgicric III	Group A (*n*=24)	122 ± 69	94 ± 55	93 ± 13	98 ± 4
	Group B (*n*=20)	115 ± 78	87 ± 47	96 ± 5	99 ± 2
	Both Groups (*n*=44)	119 ± 73	91 ± 51	95 ± 10	98 ± 4

Data are presented as mean ± standard deviation

A repeated- measures ANOVA was conducted to analyze the effects of kit type (Quicktrach II vs. Surgicric III), assessment (first vs. second), and group assignment (training order) on procedural time. The analysis revealed a significant main effect of assessment (F (1, 42) = 8.42, *p* = 0.01), indicating a significant reduction in procedural time between the first and second assessments. A significant main effect of kit type was also observed (F (1, 42) = 134.07, *p* < 0.01), with Quicktrach II being significantly faster than Surgicric III, as described before. No significant main effect of group assignment (F (1, 42) = 0.05, *p* = 0.82) was detected, suggesting that the order of simulator use did not influence procedural time.

Interaction effects between the factors were not significant, including Assessment.

×Kit (F (1, 42) = 0.21, *p* = 0.65), Group×Kit (F (1, 42) = 0.46, *p* = 0.50), and Assessment × Group×Kit (F (1, 42) = 0.08, *p* = 0.77).

**Fig. 3:**
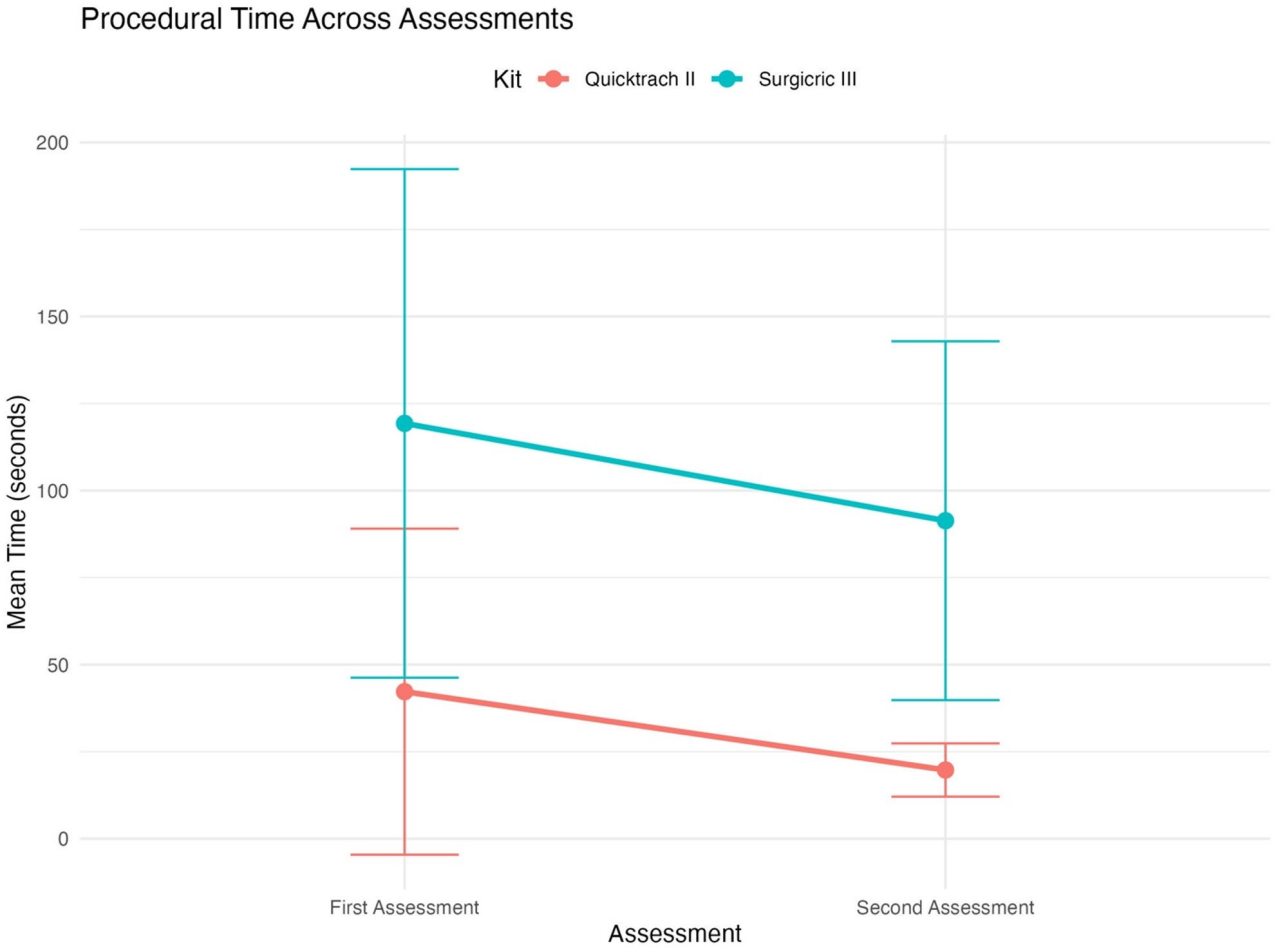
Procedural time across assessments for the two different kits in group A and B

Post- hoc comparisons did not show significant differences between groups (group A vs. B) for either Quicktrach II (*p* = 0.78) or Surgicric III (*p* = 0.65) but demonstrated that procedural time significantly improved between assessments for both kits. For Quicktrach II, the meantime decreased from 42 ± 46 s during the first assessment to 19 ± 7 s during the second assessment (*p* < 0.01). Similarly, for Surgicric III, the meantime decreased from 119 ± 73 s during the first assessment to 91 ± 51 s during the second assessment (*p* = 0.04). Figure 3 shows reduction in procedural time from first to second assessment for both kits with group A and B combined.

A repeated- measures ANOVA was conducted to investigate the effects of kit type (Quicktrach II vs. Surgicric III), assessment (first vs. second), and group assignment (Group A vs. Group B) on standardized scores.

A significant main effect of assessment was observed (F (1, 42) = 7.32, *p* = 0.01), with scores improving between the first and second assessments. No significant main effects were found for kit type (F (1, 42) = 0.80, *p* = 0.38) or group assignment (F (1, 42) = 0.15, *p* = 0.70). Additionally, no significant interaction effects were observed for Assessment × Kit (F (1, 42) = 0.01, *p* = 0.96), Group × Assessment (F (1, 42) = 0.14, *p* = 0.71), Group × Kit (F (1, 42) = 2.40, *p* = 0.13), or the three- way interaction Group × Assessment × Kit (F (1, 42) = 1.56, *p* = 0.22).

**Fig. 4:**
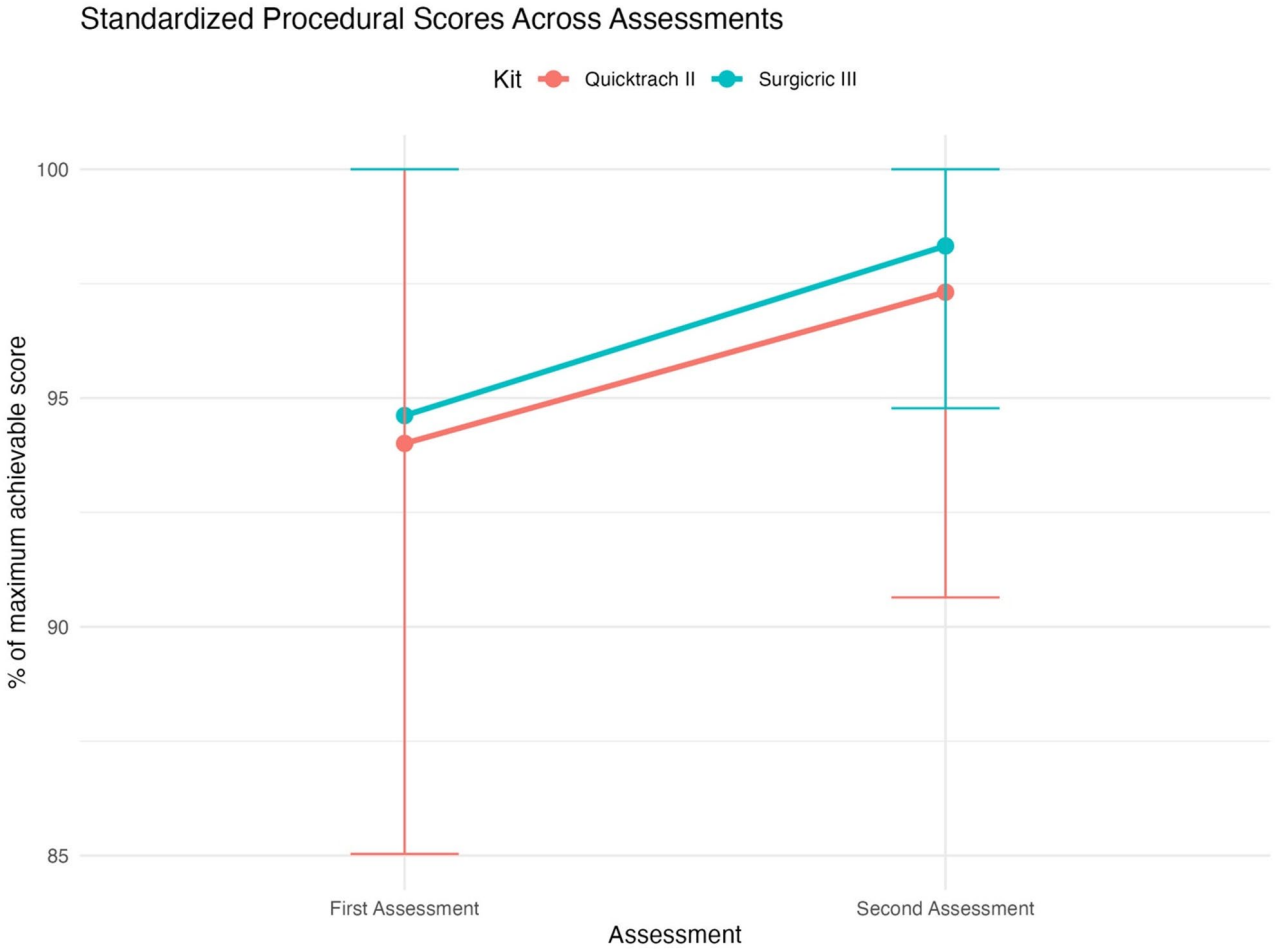
Procedural scores across assessments for both kits in groups A and B

Post-hoc analyses revealed that standardized scores for the Surgicric III kit improved significantly between the first (95 ± 10%) and second assessments (98 ± 4%; *p* = 0.04). For the Quicktrach II kit, scores also improved between the first (94 ± 9%) and second assessments (97 ± 7%), but this difference did not reach statistical significance (*p* = 0.06). No significant differences were observed between Group A and Group B (Quicktrach II *p* = 0.44, Surgicric III *p* = 0.20). Figure 4 depicts the standardized scores of first and second assessment for both kits with groups A and B combined.

### Perceived feeling of safety before and after simulation training

**Fig. 5:**
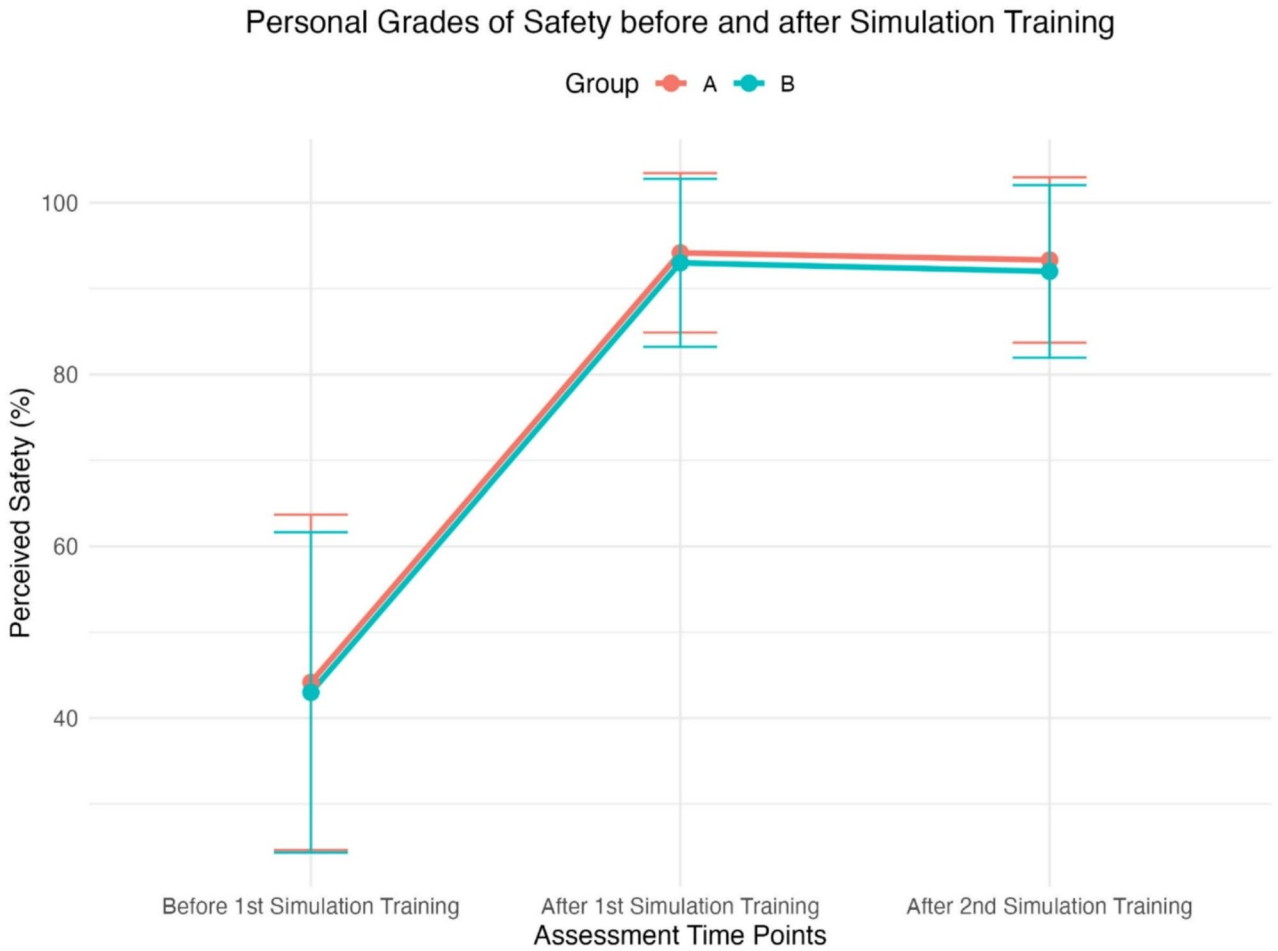
Perceived feelings of safety across assessments for both kits in groups A and B

Perceived feeling of safety significantly improved across time points (F (2, 84) = 216.70, p < 0.01), regardless of group assignment (F (1, 42) = 0.22, p = 0.64). Post- hoc analyses revealed significant increases in perceived safety from ‘Before Training’ to ‘After the First Training’ (Group A: Δ = 50.00%, p < 0.01; Group B: Δ = 50.00%, p < 0.01) and from ‘Before Training’ to ‘After the Second Training’ (Group A: Δ = 49.17%, p < 0.01; Group B: Δ = 49.00%, p < 0.01). No significant differences were observed between ‘After the First Training’ and ‘After the Second Training’ (*p* > 0.85). No interaction effect between Group and Time was found (F (2, 84) = 0.01, *p* = 1.0). The results are depicted in Table 4 and visualized in Fig. 5.

**Table 4:** Personal grades of safety in performing a percutaneous cricothyroidotomy before simulation training, after a first simulation training and after a second simulation training in *%* ranging from 0% (maximum insecurity) to 100% (maximum security)

Personal grades of safety	Group A 1) CSIM 2) 3DSIM (*n*=24)	Group B 1) 3DSIM 2) CSIM (*n*=20)
Before first ST 1 (0)	44 ± 20	43 ± 19
After first ST 1 (1)	94 ± 9	93 ± 10
After second ST 2 (2)	93 ± 10	92 ± 10

Data are presented as mean ± standard deviation.

CSIM= Conventional simulator, 3DSIM= 3D printed simulator, ST= Simulation Training

### Evaluation of simulation training

Among the 24 participants in Group A and the 20 participants in Group B, the simulation training was generally well- received and comparable between the groups. In Group A, 9 participants rated the simulation training as “very helpful” and 15 as “extremely helpful”, while in Group B, 7 participants found it “very helpful” and 13 rated it as “extremely helpful”.

Furthermore, participants of both groups evaluated the simulators’ authenticity regarding haptic (*p*=0.48) or visual characteristics within comparable grades (*p*=0.79).

## Discussion

This study offers important insights into the efficacy of simulation training of PC, utilizing two distinct simulator types, CSIM versus 3DSIM, with two different PC kits (Quicktrach II and Surgicric III). The two types of simulators demonstrated comparable efficacy in enhancing participants’ procedural performance, as evidenced by similar reductions in procedural time and perceived feelings of safety in both groups, irrespective of participant demographics.

### Differences between the Quicktrach II and Surgicric III kit

The findings of the study revealed discrepancies between the two PC kits, highlighting their differential responses to repeated simulation training. A significant reduction in procedural time was observed for both kits after the second simulation training session. Quicktrach II demonstrated shorter procedural times overall, likely attributable to its simpler design and streamlined technique. However, Surgicric III, which employs Seldinger’s technique, showed a more substantial improvement in procedural scores between assessments, suggesting that its more intricate design benefits from repeated practice to enhance precision. These findings are consistent with prior research showing that complex procedures require greater training intensity to achieve proficiency [1, 10]. 

The reduction in procedural time for Surgicric III, while significant, remained less pronounced compared to Quicktrach II. This highlights the importance of tailoring training programs to account for the technical demands of specific devices. Future studies should evaluate whether additional training sessions for kits employing Seldinger’s technique can close the gap in procedural time while maintaining high procedural accuracy [2, 11]. 

The improvement in procedural scores for Surgicric III supports the notion that repeated training can enhance participants’ proficiency, even with initially high-performance levels. Both kits enabled participants to achieve near- maximal scores during the first assessment, reflecting strong procedural accuracy across the board. However, the significant improvement observed only for Surgicric III underscores the added value of repetitive practice for mastering more complex systems.

### Impact of simulator type on training outcomes

The current study did not directly compare CSIM and 3DSIM in isolation at different timepoints, as all participants trained on both simulators in a crossover design. However, the study enabled a direct comparison between the CSIM and 3DSIM simulators after the first assessment, as participants had trained exclusively on one simulator at that time point. The results revealed no significant differences in procedural scores or procedural times between the two simulators, suggesting that both were equally effective in facilitating skill acquisition during initial training. Analysis after second assessment, when both groups had trained on both simulators, showed no differences in procedural scores, procedural times, or participants’ evaluations of the simulators’ haptic and visual characteristics, regardless of the order in which they trained on the simulators. This suggests that the sequence of training irrespective of the first simulator that was used for training, did not influence the overall effectiveness of the training.

The findings imply that both simulator types are equally effective in teaching percutaneous cricothyrotomy (PC) skills, and that the choice of simulator may not be critical to the training outcome. Previous studies have similarly found that high-fidelity simulators, like 3D- printed models, do not always provide superior training outcomes compared to traditional simulators in terms of skill acquisition [3, 8, 12]. Instead, the effectiveness of a simulator may depend more on how well it is incorporated into a broader training curriculum, the educational goals, and its cost-effectiveness [4, 13]. 

This is an important consideration for training programs, particularly in resource- limited settings where the cost and availability of high-fidelity 3D printing technology may be prohibitive. The decision to invest in 3D- printed simulators should be balanced against other factors such as the specific learning objectives, the complexity of the procedures being taught, and the available budget [5, 14]. 

### Relevance of repeated training

The significant improvement in procedural time for both kits, observed after the second simulation training, highlights the importance of repeated practice in mastering HALO skills. This is consistent with the broader literature on SBME, which emphasizes the role of deliberate practice in skill acquisition and retention [6, 7]. 

### Study limitations

Although this study was highly standardized, randomized, and blinded to reduce bias, several limitations should be acknowledged. First, the single-center design and relatively small sample size limit generalizability, despite meeting the calculated sample size requirement. The study population, primarily consisting of anesthesiologists, internal medicine physicians, and a small group of medical students, may not fully represent all healthcare providers likely to perform PC clinically.

The absence of a pre-training baseline procedural assessment prevents definitive attribution of improvements solely to the simulation intervention, as familiarity with the kits might partially explain performance gains. Moreover, the study did not include a non-simulation control group, limiting conclusions regarding the absolute impact of simulator training versus other educational interventions (e.g., theoretical instruction alone).

Additionally, long-term skill retention and transfer of skills to clinical practice were not assessed, and further longitudinal studies are necessary to clarify this relationship. Although porcine models provide a valuable approximation of human anatomy and are frequently used in simulation studies [15, 16], they cannot fully replicate human tissue complexity and patient-specific variability encountered in real-world scenarios.

Nonetheless, this study is the first to directly compare CSIM and 3DSIM using two widely available PC kits under highly standardized conditions, providing valuable insights for educators and clinicians seeking effective and practical training solutions.

## Conclusion

This study demonstrates that both conventional and 3D-printed simulators are equally effective in improving procedural performance and subjective safety perceptions for PC training, emphasizing the value of repeated hands-on practice. While procedural times improved significantly for both Quicktrach II and Surgicric III kits, Quicktrach II offered faster performance overall, whereas Surgicric III, employing a more complex technique, showed greater improvement upon repeated practice. Given the equivalence of the two simulator types in terms of educational outcomes, training programs may flexibly select a simulator based on factors such as cost, availability, and institutional resources. Future research should address the durability of these training effects and their translation to real clinical environments.

## Supplementary Information

Below is the link to the electronic supplementary material.

Supplementary Material 1

Supplementary Material 2

## Data Availability

The data that support the findings of this study are not openly available due to reasons of sensitivity and are available from the corresponding author upon reasonable request. Data are located in controlled access data storage at University Hospital Cologne, Germany.
