# Supplementary material for: Comparing conventional versus 3D printed simulators for simulation training of emergency percutaneous cricothyrotomy with two different kits: a randomized controlled trial
Source: 3D Print Med. 2026 Feb 2;12:5. doi: 10.1186/s41205-026-00315-z (PMC12930934; doi:10.1186/s41205-026-00315-z)
Supplement: Supplementary file 1 — Supplementary Material 1 [file 41205_2026_315_MOESM1_ESM.docx]

**SDC Table 1.** Scoring sheet for assessment of effectiveness of simulation training of percutaneous cricothyrotomy with the Quicktrach II kit.

| **Procedure** | **Points** |
| --- | --- |
| 1. Palpation of landmarks | 1 |
| 2. Correct choice of puncture site | 1 |
| 3. Stabilization of trachea in midline (between thumb and index finger) | 1 |
| 4. Puncture of cricoid membrane in 90° angle | 1 |
| 5. Advancement of cannula in 45° angle caudally until reaching the stopper | 1 |
| 6. Aspiration of air (control of correct position) | 1 |
| 7. Removal of stopper | 1 |
| 8. Further advancement of plastic cannula | 1 |
| 9. Removal of metal cannula | 1 |
| 10. correct position of cannula | 1 |
| 11. Coniotomy completed in given time frame | 1 |
| **Total procedure points (0-11 points)** |  |
| Time required for percutaneous cricothyroidotomy (minutes) |  |
