# Supplementary material for: Comparing conventional versus 3D printed simulators for simulation training of emergency percutaneous cricothyrotomy with two different kits: a randomized controlled trial
Source: 3D Print Med. 2026 Feb 2;12:5. doi: 10.1186/s41205-026-00315-z (PMC12930934; doi:10.1186/s41205-026-00315-z)
Supplement: Supplementary file 2 — Supplementary Material 2 [file 41205_2026_315_MOESM2_ESM.docx]

**SDC 2.** Scoring sheet for assessment of effectiveness of simulation training of percutaneous cricothyrotomy with the Surgicric III kit.

| **Procedure** | **Points** |
| --- | --- |
| 1. Palpation of landmarks | 1 |
| 2. Correct choice of puncture site | 1 |
| 3. Stabilization of trachea in midline (between thumb and index finger) | 1 |
| 4. Puncture of cricoid membrane in 90° angle | 1 |
| 5. Advancement of cannula in 45° angle caudally | 1 |
| 6. Aspiration o fair (control of correct position) | 1 |
| 7. Further advancement of plastic cannula | 1 |
| 8. Removal of metal cannula | 1 |
| 9. Insertion of Seldinger wire | 1 |
| 10. Seldinger wire inserted until 20cm marker (2 markings) | 1 |
| 11. Removal of introducer | 1 |
| 12. Removal of plastic cannula | 1 |
| 13. Scalpel incision 5mm each lateral of the wire | 1 |
| 14. Insertion of tracheal tube via dilator | 1 |
| 15. Removal of wire | 1 |
| 16. Blockage of tracheal cuff | 1 |
| 17. Removal of dilator | 1 |
| 18. correct position of tracheal cannula | 1 |
| 19. Cricothyroidotomy completed within given timeframe | 1 |
| **Total procedure points (0-19)** |  |
| Time required for percutaneous cricothyroidotomy (minutes) |  |
|  |  |
